# Atypical femoral fracture in patients with bone metastasis receiving denosumab therapy: a retrospective study and systematic review

**DOI:** 10.1186/s12885-019-6236-6

**Published:** 2019-10-22

**Authors:** Momoko Takahashi, Yukinori Ozaki, Rika Kizawa, Jun Masuda, Kentaro Sakamaki, Keiichi Kinowaki, Taro Umezu, Chihiro Kondoh, Yuko Tanabe, Nobuko Tamura, Yuji Miura, Takashi Shigekawa, Hidetaka Kawabata, Noriyuki Baba, Haruo Iguchi, Toshimi Takano

**Affiliations:** 1 Department of Palliative Care, Tokyo Medical and Dental University, Tokyo, Japan; 2 Department of Medical Oncology, Toranomon Hospital, 2-2-2 Toranomon, Minato-ku, Tokyo, 105-8470 Japan; 3 Department of Biostatistics and Bioinformatics, Graduate School of Medicine, The University of Tokyo, Tokyo, Japan; 4 Department of Pathology, Toranomon Hospital, Tokyo, Japan; 5 Department of Orthopedic Surgery, Saiseikai Yokohama-shi Tobu Hospital, Tokyo, Kanagawa Japan; 6 Department of Breast and Endocrine Surgery, Toranomon Hospital, Tokyo, Japan; 7 Department of Breast Oncology, Tokyo Kyosai Hospital, Tokyo, Japan; 8 Department of Medical Oncology, Sasebo Kyosai Hospital, Nagasaki, Japan

**Keywords:** Femoral fractures, Denosumab, Bone metastasis, Retrospective studiess, Systematic review

## Abstract

**Background:**

While denosumab has been shown to prevent skeletal-related events in patients with bone metastasis, there is a concern that it may cause atypical femoral fracture (AFF). While AFF has been reported in patients with osteoporosis receiving denosumab, data are scarce in the context of AFF occurring in patients with bone metastasis receiving monthly denosumab therapy.

**Methods:**

To analyze the incidence of AFF in patients with bone metastasis, we reviewed the medical records of patients who had received monthly denosumab (120 mg) treatment from May 2012 to June 2017 at any of the three participant institutions.

**Results:**

The study population consisted of 277 patients who had received a median of 10 doses (range, 1–79) of denosumab. Five patients were diagnosed as having AFF or symptomatic atypical femoral stress reaction (AFSR) needing surgical intervention, representing an incidence rate of 1.8% (95% confidence interval, 0.77–4.2). These patients had received 15, 45, 45, 46 or 47 doses of denosumab, respectively. Four of the patients had received prior zoledronic acid treatment. The results of our analysis suggested that long-term use of denosumab, especially for more than 3.5 years, and prior use of zoledronic acid were risk factors for the development of AFF.

**Conclusions:**

We found the AFF events in 5 patients (1.8%) among 277 cancer patients who had received monthly denosumab (120 mg) treatment. Long-term denosumab treatment and prior zoledronic acid treatment were identified as risk factors for the development of AFF.

## Background

Treatment with bisphosphonates (BP) and denosumab is known to reduce the frequency of skeletal-related events (SREs), such as pathologic bone fracture, spinal cord compression, need for external beam radiation or surgery to the bone, and hypercalcemia associated with cancer with bone metastasis [1–4]. Denosumab, an inhibitor of the receptor activator of nuclear factor κ B ligand (RANKL), has been demonstrated to delay the time to onset of the first SRE and lower the risk of occurrence of the first SRE, as compared to zoledronate, in patients with bone metastasis [5–11]. However, atypical femoral fracture (AFF) has been reported as a potential adverse effect of both BPs and denosumab. The absolute risk of AFF associated with BP therapy for the treatment of osteoporosis or osteopenia was 3.2 to 50 cases per 100,000 person-years, and patients receiving long-term treatment or much higher doses of BPs have been found to be at a higher risk of development of AFF [12–14].

According to the American Society for Bone and Mineral Research (ASBMR), AFF is defined as a fracture located along the femoral diaphysis between just distal to the lesser trochanter and just proximal to the supracondylar flare. ASBMR revised the diagnostic criteria for AFF in 2013, which now consist of five major criteria and four minor criteria [14]. Association with minimal or no trauma, as with a fall from a standing height or less is one of the major features. Localized periosteal or endosteal thickening of the lateral cortex at the fracture site, which is called “beaking” or “flaring”, is another major feature (Table 1).

**Table 1:** American Society for Bone and Mineral Research Task Force 2013 Revised Case Definition of AFF

To satisfy the case definition of AFF, the fracture must be located along the femoral diaphysis between just distal to the lesser trochanter and just proximal to the supracondylar flare. In addition, at least four of the five Major Features must be present. None of the Minor Features is required, but they are known to sometimes be associated with these fractures [14].	
Major features	
1: The fracture is associated with minimal or no trauma, as after a fall from a standing height or less.	
2: The fracture line originates in the lateral cortex and is substantially transverse in its orientation, although it may become oblique as it progresses medially across the femur.	
3: Complete fractures extend through both cortices and may be associated with a medial spike; incomplete fractures involve only the lateral cortex.	
4: The fracture is noncomminuted or minimally comminuted.	
5: Localized periosteal or endosteal thickening of the lateral cortex is present at the fracture site (“beaking” or “flaring”).	
Minor features	
1: Generalized increase in the cortical thickness at the femoral diaphysis	
2: Unilateral or bilateral prodromal symptoms, such as dull or aching pain in the groin or thigh	
3: Bilateral incomplete or complete femoral diaphysis fractures	
4: Delayed fracture healing	

Although the pathogenesis of AFF is not fully understood, the cause of AFF is considered to be stress or insufficient fractures that accumulate due to the suppression of bone remodeling by BP or denosumab treatment. Continuous loading of femur may develop microcracks within the bone cortex and BP or denosumab inhibit the normal osteoclastic resorption of them, which may eventually lead a fracture [15].

AFF has been reported in patients receiving denosumab 60 mg at 6-month intervals for osteoporosis [16–21]. On the other hand, no AFF events were recorded in clinical trials of monthly denosumab (120 mg) treatment for patients with bone metastasis, despite the higher and more frequent dosing [6–12]. Although Yang, et al. reported one case of clinical AFF in a patient receiving oncologic denosumab therapy from a retrospective review [22], clinical and pathological data are still lacking. We conducted this retrospective multi-center study to assess the incidence of AFF and the clinical and pathological features of patients with bone metastasis treated with denosumab at the dose of 120 mg monthly, and performed a systematic review of articles on this subject that we retrieved from a search of PubMed.

## Methods

In this multi-center retrospective study, we reviewed the medical records and pharmacy database of patients who had received monthly denosumab (120 mg) treatment for the management of bone metastasis from May 2012 to June 2017 at any of the three participant institutions. We then reviewed the clinical features and skeletal images of the patients who had developed AFF or atypical femoral stress reaction (AFSR) with “beaking” on radiographs. The definition of AFF was based on the definition coined by the ASBMR Task Force 2013, which is shown in Table 1 [14]. The fracture must be located along the femoral diaphysis between just distal to the lesser trochanter and just proximal to the supracondylar flare.

To identify the risk factors for AFF and the optimal management method for this condition in patients with bone metastasis receiving denosumab treatment, we conducted a systematic search of the literature for original articles in English published in the online PubMed database from January 2006 to November 2018. The search terms used were as follows: “atypical femoral fracture”, “denosumab”. We also reviewed the references cited in the included articles to identify additional studies. All the search results were evaluated independently by two of the authors (M.T. and Y.O.). The inclusion criteria for the articles to be reviewed were as follows: 1) study population: patients who had received denosumab for the treatment of bone metastasis; 2) method: prospective study, retrospective study, or case report; 3) language: English. Studies not fulfilling the above inclusion criteria, as also abstracts from conferences and commentary articles, were excluded. The last search was conducted on 1st February 2019.

In the analysis of the data from the medical records and pharmacy database, continuous variables were summarized as the means ± standard deviation or median with range, as appropriate. Categorical variables were summarized in terms of frequencies and percentages. In the analysis of the incidence rate of AFF, the 95% confidence intervals were estimated by the exact method, and differences between subgroups was compared by Fisher’s exact test. To evaluate the association between the duration of denosumab treatment and the occurrence of AFF events, the cumulative incidence curve was constructed by the Kaplan-Meier method. AFF event, in accordance with the definition proposed by the ASBMR Task Force 2013, and/or AFSR needing surgical intervention were diagnosed by one or more orthopedists. *P* values of less than 0.05 were considered as denoting statistical significance. Statistical analysis was performed using JMP version 12.0.1 (SAS Institute Inc., Cary, NC, USA) and SAS version 9.4 (SAS Institute Inc., Cary, NC, USA).

## Results

We analyzed the data of 277 patients, and the patient characteristics are shown in Table 2. The median age of the patients was 65 (range, 29–88) years, and the primary disease was breast cancer in the majority (57%). Other primary diseases (5%) included GIST, head and neck cancer, neuroendocrine carcinoma, thyroid cancer, salivary gland cancer, thymoma, leiomyosarcoma, gallbladder carcinoma, urothelial cancer and dermatofibrosarcoma (Table 2). Metastatic sites other than bone metastasis mainly included the lung (43%), liver (39%), lymph node (53%), etc.; other sites included the skin, pancreas, retina, ovary and salivary gland. Among the 277 patients, 56 (20%) had received prior zoledronic acid treatment. The patients had received a median of 10 doses (range; 1–79) of denosumab, and 16 (5%) patients had received more than 45 doses. The clinical features of the patients with breast cancer are shown in Table 3; the majority had hormone receptor-positive breast cancer (81%). Of the breast cancer patients, 61 (38%) had received preoperative chemotherapy, and 39 (25%) had received aromatase inhibitor treatment as adjuvant endocrine therapy; furthermore, 39 (25%) patients had received prior zoledronic acid treatment. The breast cancer patients had received a median of 18 doses (range; 1–79) of denosumab, and 15 (9%) patients had received more than 45 doses. The sites of bone metastasis are shown in Table 4. The most frequent metastatic sites were the thoracic vertebrae (44%), and 15% of the patients had femur metastasis. Other skeletal sites included the clavicle, mandible and tibial bone.

**Table 2:** Patient characteristics

	Number of patients
Characteristics	*n* = 277, n (%)
Age, median (range)	median 65 (29–88)
Gender	
Male	93 (34)
Female	184 (66)
Primary disease	
Breast cancer	159 (57)
Prostate cancer	26 (9)
Pancreatic cancer	13 (5)
Lung cancer	12 (4)
RCC	12 (4)
Gastric cancer	11 (4)
Carcinoma of unknown primary	8 (3)
Colorectal cancer	8 (3)
Esophageal cancer	5 (2)
Urothelial cancer	4 (1)
Melanoma	2 (1)
Hepatocellular carcinoma	2 (1)
Others	15 (5)
Stage at diagnosis	
I	21 (8)
II	58 (22)
III	46 (17)
IV	94 (35)
Unknown	46 (17)
Perioperative chemotherapy	
Yes	80 (29)
No	195 (70)
Unknown	2 (1)
Metastatic site	
Lung	120 (43)
Liver	107 (39)
Lymph node	147 (53)
Bone	277 (100)
Brain	28 (10)
Pleura	23 (8)
Peritoneum	20 (7)
Adrenal gland	17 (6)
Others	19 (7)
Prior zoledronic acid treatment	
Yes	56 (20)
No	167 (60)
Unknown	54 (19)
Number of doses of denosumab	median 10 (1–79)
More than 45 doses of denosumab	16 (5)

**Table 3:** Characteristics of the patients with breast cancer

	Number of patients
Characteristics	*n* = 159 (%)
Stage at diagnosis	
I	17 (11)
II	50 (31)
III	32 (20)
IV	31 (20)
Unknown	28 (17)
Subtype	
HR+ HER2-	108 (68)
HR+ HER2+	20 (13)
HR- HER2+	9 (6)
HR- HER2-	14 (9)
Unknown	8 (5)
Perioperative chemotherapy	
Yes	61 (38)
Anthracycline regimen	11 (7)
Anthracycline + taxane regimen	26 (16)
Other regimens	24 (15)
No	97 (61)
Unknown	1 (1)
Adjuvant endocrine therapy	
Aromatase inhibitor	39 (25)
Tamoxifen	29 (19)
Ovarian function suppression	22 (14)
None	22 (14)
Unknown	1 (1)
Prior treatment with zoledronic acid	
Yes	39 (25)
No	92 (60)
Unknown	23 (15)
Number of denosumab doses	median 18 (range: 1–79)
More than 45 doses of denosumab	15 (9)

**Table 4:** Sites of bone metastasis

	Number of patients
Site of bone metastasis	*n* = 277 (%)
Cervical vertebra	53 (19)
Thoracic vertebra	123 (44)
Lumbar vertebra	115 (42)
Sacral bone	38 (14)
Costal bone	77 (28)
Sternum	43 (16)
Pelvis	100 (36)
Femur	41 (15)
Humerus	19 (7)
Scapula	21 (8)
Cranium	14 (5)
Others	14 (5)

Among the 277 patients, five patients were diagnosed as having AFF and/or AFSR needing surgical intervention, representing an incidence rate of 1.8% (95% CI: 0.77–4.2). The details of the clinical features of these five patients are shown in Table 5.

**Table 5:** Clinical features of the AFF cases identified in this retrospective study

Case No.	Primary disease	Site of metastasis	Prior zoledronic acid treatment	Doses of denosumab	Diagnosis	Operation
1	Breast Ca.	Bone	+	45	Lt. AFF Rt. AFSR	+ -
2	Breast Ca.	Bone, Skin, Lung	+	47	Lt. AFF Rt. femur meta.	+ -
3	Breast Ca.	Bone	+	45	Lt. AFSR Rt. AFSR	+ +
4	Breast Ca.	Bone, Lung, LN	+	46	Lt. AFF Rt. AFSR	+ +
5	NSCLC	Bone, Lung, LN, CNS	–	15	Lt. AFF Rt. intact	+ -

### Case 1

**Fig. 1:**
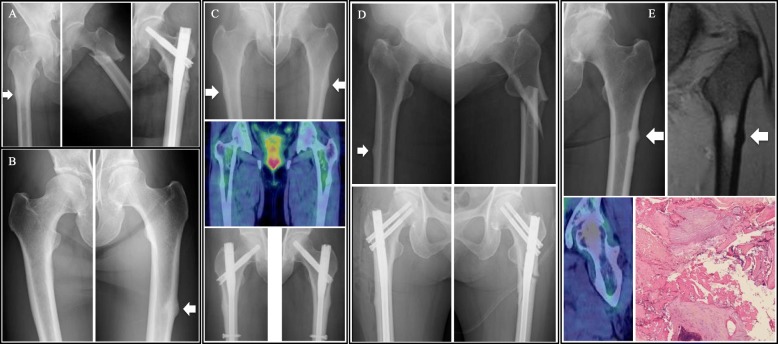
Radiological findings of atypical femoral fracture. **a** X-ray images of Case 1. White arrow shows atypical femoral stress reaction (AFSR) on the right femur. X-ray of the left femur at the time of the AFF event and after intramedullary nail surgery. **b** X-ray images of Case 2. White arrow shows AFSR in the left femur. **c** X-ray and PET/CT images of Case 3. The upper images show bilateral AFSR and the middle image shows no metastasis in either femur. The lower images are x-rays obtained after prophylactic intramedullary nail fixation surgery. **d** X-ray images of Case 4. The upper images show left AFF and right AFSR. The lower images are X-rays after intramedullary nail fixation surgery on both sides. **e** X-ray, MRI and PET/CT images of Case 5. The upper left image is an x-ray showing left AFSR and upper right image is an MR image of the same region. The lower left image is a PET/CT image showing no evidence of malignancy and the lower right image is hematoxylin and eosin staining of fracture tissue from Case 5 (× 400). The section shows fragmented bone tissue, fibrous tissue, calcification, and a little bone marrow tissue

A patient was diagnosed as having invasive ductal carcinoma of the left breast. The patient underwent left mastectomy and axillary lymph node (LN) dissection, and analysis of the specimen revealed that the cancer was estrogen receptor (ER)- positive, progesterone receptor (PR)- positive, and HER2-negative. The pathological stage was pTxN1M0, and the patient received 6 cycles of adjuvant cyclophosphamide, methotrexate and fluorouracil chemotherapy, followed by adjuvant hormone therapy with tamoxifen for 3 years. Multiple bone metastases (cervical, thoracic and lumbar vertebrae) were detected and treatment was initiated with an aromatase inhibitor, anastrozole, and zoledronic acid at 13 years after initial diagnosis. Disease progression was detected and administration of exemestane was initiated. At 16 years after initial diagnosis, zoledronic acid was switched to denosumab. Positron emission tomography/computed tomography (PET/CT) showed a new lesion in a thoracic vertebra and the hormone therapy was switched to fulvestrant. Capecitabine administration was initiated due to disease progression. Subsequently, the patient fell and was diagnosed as having left AFF after 45 doses of denosumab and underwent intramedullary nail surgery (Fig. 1a). The x-ray also showed right AFSR. CT performed before the AFF event showed no bone metastasis in the femur. The patient received the last dose of denosumab treatment at 20 years after initial diagnosis and continued to receive chemotherapy at cut-off date.

### Case 2

A patient who underwent partial resection of the right breast and axillary LN dissection for breast cancer; the tumor was ER/PR-negative and HER2-negative. The patient received radiation therapy at 50 Gy, but no adjuvant chemotherapy. At 9 years after initial diagnosis, local recurrence was detected, which was resected, followed by no adjuvant treatment. Local recurrence was detected in the left breast and left mastectomy was performed. Metastatic lesions were seen in the skin and sternum, and the patient was initiated on treatment with an LH-RH analog + tamoxifen and a bisphosphonate at 2 years after the second surgery. The bisphosphonate was switched into denosumab. The patient was started on an LH-RH analog + anastrozole after detection of a lung metastasis, and on exemestane + everolimus after detection disease progression. The patient noticed pain in the left hip, but hip x-ray taken at a neighborhood clinic revealed no abnormalities. The patient lost his/her balance and the hip pain worsened. PET/CT revealed no abnormal uptake in the area. Radiographs of left femur showed thickening of the lateral cortex. The patient was diagnosed as having left AFF and underwent prophylactic surgery after 47 doses of denosumab (Fig. 1b) at 18 years after initial diagnosis. MRI revealed bone metastasis in the right femur. After the AFF event, the patient stopped to receive denosumab treatment and continued to received hormone therapy. The patient died due to progression of breast cancer at 20 years after initial diagnosis.

### Case 3

A patient was diagnosed as having invasive ductal carcinoma of the right breast. Partial resection of the right breast with axillary LN dissection was performed, and on histopathology, the tumor was staged as pT1N1M0. The tumor was ER/PR-positive, and negative for HER2 overexpression. The patient was followed up with no adjuvant treatment. Tumor recurrence with bone metastasis in the 12th thoracic vertebra (Th12) was detected at 10 years after initial diagnosis, and radiation therapy at 39 Gy/13 Fr was performed. The patient was also treated with arimidex and a bisphosphonate, and treatment with denosumab 120 mg monthly was started. Arimidex was changed to tamoxifen because new bone lesions were detected. At 15 years after initial diagnosis, the patient complained of persistent hip pain after a fall, but PET/CT revealed no abnormal uptake in the area. An x-ray of the hip joint showed lateral femoral thickening on both sides, and the patient was diagnosed as having bilateral symptomatic AFSR after 45 doses of denosumab. Prophylactic intramedullary nail fixation surgery was performed (Fig. 1c). Denosumab administration was resumed at 2 months after the operation. No other skeletal event was occurred and the patient continued to receive hormone therapy at cut-off date.

### Case 4

A patient was diagnosed as having invasive ductal carcinoma of the left breast; the clinical stage was cT3N1M0 and the tumor was positive for ER, negative for PR, and positive for HER2 overexpression. The patient received neoadjuvant chemotherapy involving 4 cycles of cyclophosphamide plus epirubicin, followed by paclitaxel and trastuzumab. Partial resection of the left breast and axillary LN dissection were performed, and the pathological stage of the tumor was pT1N1M0. The patient received radiation therapy at 60 Gy and trastuzumab every 3 weeks for a year. Letrozole and zoledronic acid were also administered. At 4 years after initial diagnosis, tumor recurrence was detected in the sternum and parasternal lymph nodes, and the patient was treated with trastuzumab plus toremifene. Zoledronic acid was switched into denosumab 120 mg monthly. Several regimens for further relapses followed, including capecitabine + lapatinib, pertuzumab + trastuzumab + docetaxel, trastuzumab emtansine, and pertuzumab + trastuzumab + vinorelbine. The patient developed pain in the left hip joint and difficulty in walking at 8 years after initial diagnosis, however, an MRI of the pelvis revealed no abnormalities. MRI of the lumbar spine showed a lesion which was suspected as being a metastasis. Also, a high intensity area was seen in the proximal aspect of the thigh bone and metastasis was suspected. There was no pain in those lesions and a corset was prescribed for prevention of fracture. The patient accidentally turned over and was diagnosed as having left AFF after 46 doses of denosumab treatment. Intramedullary nail fixation was performed and histopathology showed no evidence of malignancy. An x-ray showed right lateral femoral thickening and stress reaction was suspected. Since load on the right femur was not recommended in this situation, prophylactic intramedullary nail fixation surgery of the right femur was performed. Denosumab administration was resumed at 4 months after the operation (Fig. 1d). No other skeletal event was occurred and the patient was admitted to hospice at 9 years after diagnosis.

### Case 5

A patient with a past history of breast cancer who was diagnosed as having with ALK mutation-positive NSCLC, stage cT3N3M0. The patient received concurrent chemoradiotherapy with curative intent, with 4 cycles of cisplatin and vinorelbine. At 1 year after diagnosis, the patient was diagnosed as having relapse with lung metastases which were positive for ALK mutation, and was started on crizotinib treatment. PET/CT showed intense uptake in Th12 and monthly denosumab treatment at 120 mg was started. The patient had a past history of breast cancer and had previously received alendronate treatment for osteoporosis prior to the denosumab treatment. After 3 years after initial diagnosis, the patient felt pain in the left hip. Since the pain gradually became worse, MRI was performed, which showed left AFSR. No abnormal FDG uptake in the area was detected on PET/CT (Fig. 1e). Although orthopedic surgeons recommended prophylactic surgery for the left AFSR, the patient did not wish to undergo the surgery. However, the patient was diagnosed as having AFF in the left femur at 5 months after the diagnosis of left AFSR, after 15 doses of denosumab, and underwent open reduction and internal fixation surgery. Histopathological examination revealed fragmented bone tissue and fibrous tissue, consistent with fracture, and there was no evidence of malignancy (Fig. 1e). After the surgery, the patient did not resume denosumab treatment. The patient continued to receive crizotinib treatment without any other skeletal event at cut-off date.

### Analysis of medical records and pharmacy database

The incidence rates of AFF in the 277 patients is shown in Table 6. Fisher’s exact test was used to evaluate the relationship between potential risk factors and the risk of AFF, which showed that the number of doses of denosumab and prior zoledronic acid treatment were correlated with the risk of development of AFF (*p* = 0.0057 and 0.0151, respectively) (Table 6). Similar results of Fisher’s exact test were obtained in the breast cancer patient group. A larger number of denosumab doses and prior zoledronic acid treatment were correlated with the development of AFF in the breast cancer patients (*p* = 0.01 and 0.0062, respectively). (data not shown).

**Table 6:** Fisher’s exact test for AFF in univariate analysis

	Category	Incidence rate (95% CI)	*p*-value
All		0.018 (0.006–0.042)	
Primary disease	Breast	0.025 (0.007–0.064)	0.3968
	Other	0.008 (0–0.046)	
Stage at diagnosis	Stage 1–2	0.013 (0–0.069)	1
	Stage 3–4	0.014 (0.002–0.051)	
Gender	Female	0.027 (0.009–0.063)	0.1713
	Male	0 (0–0.039)	
Age	< 65	0.023 (0.005–0.065)	0.6716
	> = 65	0.014 (0.002–0.049)	
Metastatic site: liver	No	0.03 (0.01–0.068)	0.1601
	Yes	0 (0–0.034)	
Metastatic site: lung	No	0.013 (0.002–0.045)	0.6548
	Yes	0.025 (0.005–0.072)	
Metastatic site: lymph node	No	0.023 (0.005–0.066)	0.669
	Yes	0.014 (0.002–0.049)	
Metastatic site: femur	No	0.021 (0.007–0.049)	1
	Yes	0 (0–0.086)	
Number of denosumab doses	< 10	0 (0–0.027)	0.0605
	> = 10	0.035 (0.012–0.081)	
Number of denosumab doses	< 25	0.005 (0–0.025)	0.0057
	> = 25	0.074 (0.021–0.179)	
Prior zoledronic acid treatment	No	0.006 (0–0.033)	0.0151
	Yes	0.071 (0.02–0.173)	

**Fig. 2:**
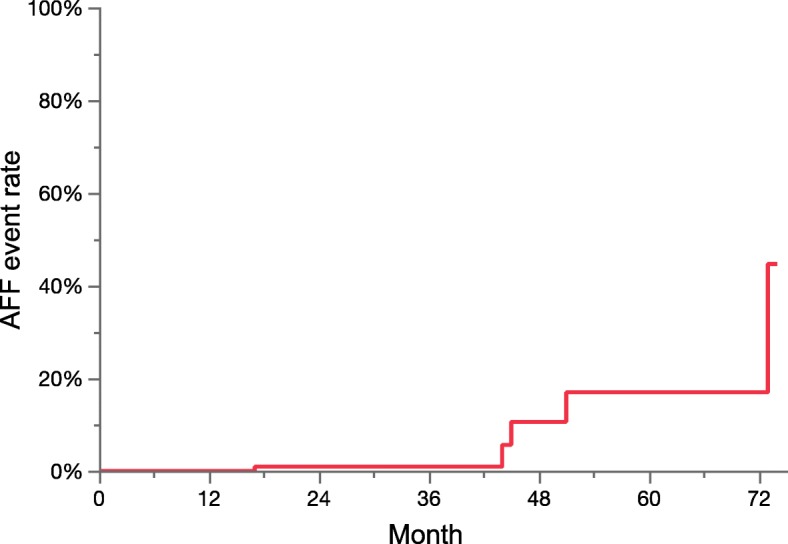
Cumulative frequency curve of atypical femoral fracture events in patients with bone metastasis receiving denosumab 120 mg monthly

The cumulative incidence curve for the association between the duration of denosumab treatment and the occurrence of AFF events is shown in Fig. 2. The curve suggests that patients who had received denosumab treatment for more than 42 months were at a higher risk for AFF events than those who had received the drug for shorter durations.

### Systematic review

**Fig. 3:**
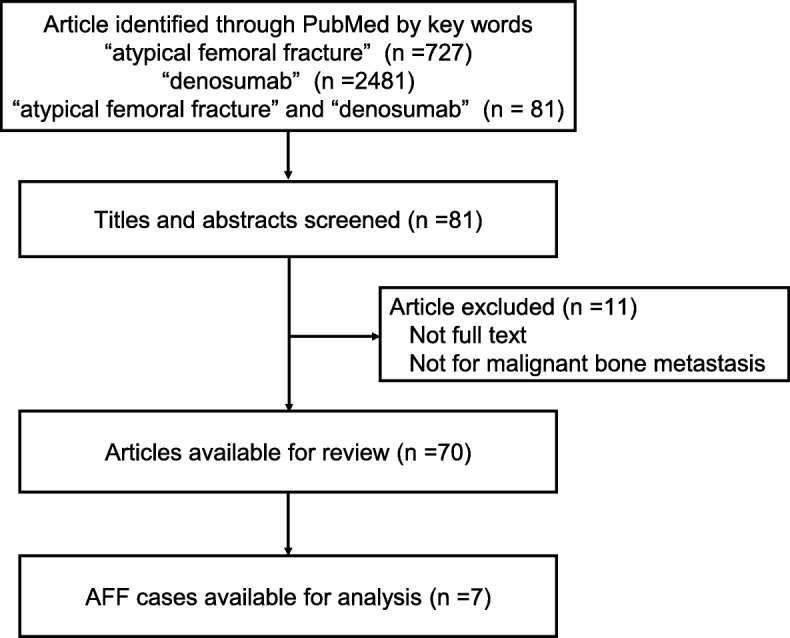
Flow diagram of the literature search and study selection process

We conducted a search of PubMed and performed a systematic review to identify the risk factors for AFF and the optimal management method for this condition. The search terms used were “atypical femoral fracture” and “denosumab”. Figure 3 shows a flow diagram of the literature search and study selection process. The number of articles identified through PubMed using the keywords “atypical femoral fracture and denosumab” was 81. Articles which were not full-text articles or did not refer to denosumab use for bone metastasis were excluded. Two oncologists individually reviewed 70 articles and found that 7 cases of AFF in patients receiving denosumab treatment for bone metastasis were available for analysis. Table 7 shows the clinical features of these cases [22–27]. The primary disease in 5 of these 7 cases was breast cancer; the other two cases had thyroid cancer or prostate cancer. The majority of the patients were female. Four patients had received prior bisphosphonate therapy with drugs such as zoledronic acid, alendronate, pamidronate or risedronate. The duration of denosumab treatment at the time of the AFF event was 18 to 54 months, and three of the patients (43%) had received more than 42 monthly doses.

**Table 7:** Clinical features of AFF cases published in the literature

Journal	Age/Gender	Primary disease	Exposure to other BMA	Duration of denosumab treatment	Diagnosis	Operation (treatment)
Yang et al. The oncologist (2017)	70/F	Breast Ca.	Alendronate, Risedronate	23 months	Rt. AFF	Intramedullary rod placement
Ota et al. Breast Cancer (2017)	73/F	Breast Ca.	Zoledronic acid	54 months	Bilateral AFF	
Sugihara et al. Clin Nucl Med (2018)	59/F	Breast Ca.	None	4 years (48 months)	Rt. impending AFF or AFSR	
Koizumi et al. Clin Nucl Med (2017)	53/F	Thyroid Ca.	Bisphosphonate	1.5 years (18 months)	Lt. impending AFF	
Tateiwa et al. Journal of orthopaedic surgery (2017)	52/F	Breast Ca.	Pamidronate, Zoledronate	2 years and 3 months (27 months)	Bilateral AFF	Intramedullary nail was placed
Austin et al. Acta Orthopedica (2017)	75/F	Breast Ca. NSCLC	None	2 years (24 months)	Bilateral AFF	Intramedullary fixation
	86/M	Prostate Ca.	None	3.5 years (42 months)	Rt. AFF	

## Discussion

The risk for AFF associated with BP and denosumab treatment remains a concern, despite the proven efficacy of these drugs in preventing SREs. Although patients with incomplete AFF often complain of thigh pain, the condition can easily go undiagnosed or be misdiagnosed before it becomes a complete fracture. Screening for AFF with plain radiographs in high-risk patients receiving denosumab therapy may help prevent complete AFF and encourage early prophylactic surgery. It is also important to note that AFF is often bilateral.

The causative relationship with BPs and denosumab has not been completely elucidated, however, over-suppression of bone turnover is the likely cause of the pathologic fracture [28]. This effect extends to the remodeling of the callus formed at the fracture site, making treatment of AFF sometimes challenging, with higher rates of delayed union and nonunion [14, 29]. Treatment guidelines for AFF have not been established and whether prophylactic surgery or conservative treatment should be chosen for incomplete AFF remains a matter of debate. Yet, non-operative treatment does not appear to be a reliable option, as the treatment fails in nearly half of the cases, eventually necessitating surgery. Koh et al. reported a total of 159 incomplete AFFs initially treated non-operatively, of which only 45% healed after the non-operative treatment [28]. A total of 47% of the cases were eventually treated operatively; 20% showed progression to complete fracture within a mean time from incomplete to complete fracture of 6 months; 27% were treated for pain relief. The remaining 8% showed failure of healing during follow up. Non-operative treatments include partial weight bearing, discontinuation of BPs, prescription of calcium and vitamin D, and teriparatide therapy, which may often be unsuitable for patients with metastasis.

We reviewed the data of the five patients who developed AFF and/or AFSR needing surgical intervention among the 277 patients who had received monthly denosumab treatment at 120 mg (corresponding to an incidence rate of 1.8%). The age range was 45 to 67 years, and cases 1–4, with breast cancer, had received more than 45 doses (42 months) of denosumab treatment for bone metastasis and had a history of prior zoledronic acid treatment. We could not find any obvious risk factor for AFF in case 5. There has been only one report of AFF in the context of higher-dose/more frequent denosumab therapy for metastatic bone disease. Yang, et al. reported an incidence rate of AFF in this context of 0.4% (1/253) and of AFSR of 4.5% (3/66) [22]. Although this previous report did not evaluate the risk factors for AFF as there was only a single event, our analysis in this multi-center retrospective study suggested that prior zoledronic acid treatment and long-term use of denosumab treatment, especially more than 45 doses (3.5 years), were correlated with the risk of development of AFF. This is compatible with the report that a longer duration of bisphosphonate therapy or denosumab for osteoporosis is an important risk factor for AFF events. We reviewed the data of 277 patients, a larger number of patients as compared to all previous reports. This is clinically meaningful inasmuch as this study suggests that the risk of this adverse event with denosumab treatment may have increased with the increased survival time of cancer patients due to the development of novel anti-cancer drugs as recently demonstrated, for example, for breast cancer patients.

We systematically reviewed the data to identify the risk factors for AFF and the optimal management method for this condition. Only one article has reported the incidence rate of AFF in this context (only one patient developed AFF in that study) [22]. We reviewed 70 articles and found seven cases of AFF among the patients with bone metastasis who had received denosumab treatment. No study until now has evaluated the risk factors for AFF events in patients receiving denosumab treatment for bone metastasis. The duration of denosumab treatment was more than 42 months in about a half of these seven cases. Also, more than a half of the cases had a history of previous bisphosphonate therapy, including with zoledronic acid, which was compatible with our results.

Our study had limitations. First, this was a retrospective study, therefore, selection bias could have influenced the incidence rate. The final incidence of 1.8% of the AFF rate is too small to draw conclusion. However, this was the largest retrospective study until date and the first study to evaluate the risk factors for AFF in patients with bone metastasis. Second, we did not perform screening skeletal imaging tests or vitamin D levels before denosumab administration, which means the baseline skeletal condition was not evaluated. Third, although we reviewed the history of prior use of zoledronic acid, the duration of treatment with zoledronic acid or other bisphosphonates was not reviewed. Forth, publication bias in the systematic review could not be excluded and we could not conduct statistical analysis due to limited statistical data.

In conclusion, this multi-center retrospective study showed that the incidence rate of AFF or symptomatic AFSR in the 277 patients who had received monthly denosumab (120 mg) treatment for bone metastasis was 1.8%. Our analyses also suggested that long-term use of denosumab and prior use of zoledronic acid were risk factors for AFF events. We are aware that this is a retrospective study and there are many limitations. Further accumulation of data is needed for more precise evaluation of AFF in patients with bone metastasis receiving denosumab therapy.

## Conclusions

This multi-center retrospective study found the incidence rate of AFF events was 1.8% among 277 cancer patients who had received monthly denosumab treatment. Long-term denosumab treatment and prior zoledronic acid treatment were identified as risk factors for the development of AFF.

## Data Availability

The datasets used and analyzed during the current study are available from the corresponding author on reasonable request.

## Abbreviations

AFF: Atypical femoral fracture
AFSR: Atypical femoral stress reaction
ASBMR: American Society for Bone and Mineral Research
BPs: Bisphosphonates
ER: Estrogen receptor
FDG: Fluorodeoxyglucose
HER2: Human epidermal growth factor receptor type2
LH-RH: Luteinizing hormone-releasing hormone
LN: Lymph node
MRI: Magnetic resonance imaging
PET/CT: Positron emission tomography/computed tomography
PR: Progesterone receptor
RANKL: Receptor activator of nuclear factor κ B ligand
SREs: Skeletal-related events
Th: Thoracic vertebra
